# Secular Trends in Prevalence of Overweight and Obesity among Adults in Rural Tianjin, China from 1991 to 2011: A Population-Based Study

**DOI:** 10.1371/journal.pone.0116019

**Published:** 2014-12-29

**Authors:** Xianjia Ning, Changqing Zhan, Yihe Yang, Li Yang, Jun Tu, Hongfei Gu, Ta-Chen Su, Jinghua Wang

**Affiliations:** 1 Department of Epidemiology, Tianjin Neurological Institute, Tianjin, China; 2 Department of Neurology, Tianjin Medical University General Hospital, Tianjin, China; 3 Department of Public Health, Feinberg School of Medicine, Northwestern University, Chicago, Illinois, United States of America; 4 Department of Neurology, Tianjin Dagang Oilfield General Hospital, Tianjin, China; 5 Cardiovascular Center and Department of Internal Medicine, National Taiwan University Hospital, Taipei, Taiwan; Medical College of Soochow University, China

## Abstract

**Objectives:**

Obesity is associated with cardiovascular diseases and has become the main public health issue in western countries and urban China. However, the prevalence and secular trends of obesity in rural China are currently unknown. The aim of this study was to investigate secular trends in the prevalence of overweight and obesity among rural adults in northern China between 1991 and 2011.

**Method:**

The prevalence of overweight and obesity was assessed in adults aged 35–74 years living in a rural area in northern China by comparing two surveys that were conducted in 1991 and 2011, respectively.

**Result:**

The age-adjusted prevalence of overweight increased from 24.5% in 1991 to 42.0% in 2011, and the prevalence of obesity increased from 5.7% in 1991 to 19.6% in 2011. Over the 21-year period, there were significant increases in the prevalence of overweight and obesity for both men and women in all age groups; however, the greatest increase was observed in men aged 35–44 years, with an 10.3-fold increase in obesity prevalence. The prevalence of obesity increased significantly in all risk factors categories, including education levels, blood pressure categories, diabetes previous history, current smoking situation and alcohol drinking situation over the past 21 years overall (*p*<0.05). The greatest increase in obesity prevalence appeared among those who consumed alcohol (increased by 8.0-fold). Next, there was a 5.3-fold increase in the prevalence of obesity in illiterate residents.

**Conclusion:**

The prevalence of overweight and obesity has increased rapidly among rural adults in Tianjin over the past 21 years, with the most dramatic increase observed in young men. Therefore, the burden of obesity should serve as a call for action.

## Introduction

Overweight and obesity are significant clinical and public health burdens worldwide, with 205 million men and 297 million women reported to be obese in 2008 [1]. Evidence from China’s first comprehensive nutrition and health survey in 2002 indicated that the prevalence rates of overweight and obesity among adults were 23% and 7%, respectively [2]. This high prevalence of obesity is associated with an increased mortality rate due to obesity-related diseases, including cardiovascular diseases, stroke, and cancer [1], [3]–[5].

Over the past decade, the prevalence of obesity in developed countries either remained stable or significantly decreased [6]–[10]; however, obesity prevalence continues to increase in developing countries, including China [11]. Along with rapid economic development, there have been great changes in lifestyle and diet in China, especially in rural areas. More than half of the Chinese population lives in rural areas, and they tend to have poor medical insurance, low educational levels, and low income. As these areas undergo urbanization, it is critical to control several important risk factors related to chronic non-infectious diseases. The long-term trends and latest prevalence rates of overweight and obesity in rural China have not yet been reported. In our previous studies, we explored the trends in the prevalence of hypertension and incidence of stroke in a rural population [12]–[14]. In this study, we aimed to investigate the trends and current data regarding the prevalence of overweight, obesity, and associated-cardiovascular risk factors among adults in rural China from 1991 to 2011.

## Methods

### Study population

The study population was from the Tianjin Brain Study, a population-based stroke surveillance study in a township in Tianjin, China in 1985. The sampling method was reported in the previous study [12]. There were 18 administrative villages, and 95% of residents were farmers with relatively low levels of education and income. The primary source of income was grain production; annual per capita income was <100 USD in 1991 and <1000 USD in 2010 [11]. The illiteracy rate in this population was 30% in men and 40% in women for residents aged 35–74 years in 1991 [12]. Few residents were covered by national medical insurance before 2008 [15]. Because of the household registration system and traditional habits, the residents typically lived in the same village for generations, except married daughters or those going to the universities. Ten percent of men aged 35 to 64 years usually moved to the cities for seasonal work and returned home for harvests and festivities at least 4 times a year.

We compared the trends and prevalence of overweight and obesity between 2 surveys conducted in adults aged 35–74 years, according to the same methods of the previous study. Briefly, the first survey was conducted in 1991, and the second one was conducted in 2011 [12]. First, we divided all villages into 3 strata by geographical location: east, south, and north. We randomly sampled 2 villages from each stratum. Using a stratified cluster, random sampling method, we then selected residents aged ≥15 years without previous coronary heart disease and stroke from these 6 selected villages to participate in the survey. The population recruited in 1991 consisted of 3,007 residents aged 35–74 years; 2,349 residents (78.1%) participated in this health survey. The health survey collected data on blood pressure, body weight, and height and also included structured questionnaires on histories of heart diseases, stroke, and cancers; alcohol consumption and smoking habits; and hypertensive management. Excluding 153 subjects with incomplete data, 2,196 individuals were analyzed in this survey.

The survey conducted in 2011 originally consisted of 2,473 residents aged 35–74 years without history of coronary disease or stroke. The participants were selected from the same villages as those included in the previous survey. Of these, 1,996 residents completed both the questionnaire and physical examination (response rate of 80.7%). Excluding 57 individuals with no physical examination data, the final number of residents included in the 2011 survey was 1,939.

The ethics committee of Tianjin Medical University General Hospital approved the study, and written informed consent was obtained from all residents during recruitment.

### Survey methods

By prior appointments made in local health centers, face-to-face interviews and physical examinations were conducted by locally trained research staff guided by epidemiological professionals. The interviewers used a questionnaire designed to collect the following information: demographic data, including name, sex, date of birth, and educational level; history of hypertension, diabetes, cardiovascular disease, and stroke; family history (parents and siblings) of hypertension, diabetes, cardiovascular disease, and stroke; and behavioral habits (smoking and alcohol consumption). Smoking was defined as the use of at least 1 cigarette/daily for 1 year. Drinking was defined as consuming Chinese liquor at least once per week consecutively for 1 year. The physical examination included measurements of blood pressure, body height, and body weight.

### Measurements

Surveys were conducted from July to September in both 1991 and 2011. Body weight and height were measured to the nearest tenth of a kilogram by trained local physicians using a standard protocol and techniques, with subjects wearing light clothing and no shoes. Height was measured to the nearest tenth of a centimeter with a stadiometer, with subjects wearing no shoes. All investigators successfully completed a training program based on both questionnaire and measurement methods. Body mass index was calculated as weight (in kilograms) divided by the square of height (in meters). According to standard criteria in Chinese adults, overweight and obesity were defined as body mass indices of 24.0–27.9 kg/m^2^ and ≥28.0 kg/m^2^, respectively [16].

Hypertension was defined as an average SBP≥140 mmHg and/or an average DBP≥90 mmHg, or self-reported hypertension with or without current use of antihypertensive medication. Blood pressure was defined as follows: normal (SBP<120 mmHg and DBP<80 mmHg); prehypertension (SBP = 120–139 mmHg and/or DBP = 80–89 mmHg); stage I hypertension (SBP = 140–159 mmHg and/or DBP = 90–99 mmHg); and stage II hypertension (SBP≥160 mmHg and/or DBP≥100 mmHg) [17].

Diabetes was defined according to self-reported the previous history of diabetes or current use of antidiabetic medication.

### Statistical analysis

The age-adjusted prevalence of overweight and obesity and 95% confidence intervals were calculated using direct standardization, with the 2000 world population used as the standard [18]. Continuous variables were presented as the mean ± standard deviation, and values were compared between 1991 and 2011 using the Student’s *t*-test. Categorical variables were presented as frequencies and the 95% confidence interval, and values were compared between 1991 and 2011 using the chi-square test. The prevalence of overweight and obesity was analyzed by age group (35–44, 45–54, 55–64, and 65–74 years) and sex. Educational level was divided into 4 groups according to the years of education received: illiterate, <6 years, 6–8 years, and ≥9 years. The odds ratios of hypertension to overweight and obesity were calculated by multivariate regression analyses, adjusted by education level, smoking, and alcohol consumption. We used multivariate regression to estimate age adjusted odds ratios and 95% confidence intervals for overweight and obesity. A *p* value<0.05 was considered statistically significant. SPSS for Windows (version 13.0; SPSS Inc., Chicago, IL, USA) was used for analyses.

## Results

### Characteristics of the study population for the two surveys

In 1991, there were 2,196 residents aged 35–74 years without a history of cardiovascular and cerebrovascular diseases. There were 1,939 corresponding participants included in the 2011 survey. The prevalence of illiteracy declined from 35.6% in 1991 to 14.1% in 2011. The frequency of self-reported diabetes and alcohol consumption increased, whereas the rate of smoking significantly decreased from 1991 to 2011. Hypertension prevalence increased by 30%, with 40% incidence in 1991 and 52% incidence in 2011 (*p*<0.001; Table 1).

**Table 1:** Demographic characteristics of the participants between 1991 and 2011.

Characteristics	Men	Women		Total	
	1991	2011	1991	2011	1991	2011
n (%)	1032 (47.0)	865 (44.6)	1164 (53.0)	1074 (55.4)	2196 (100.0)	1939 (100.0)
Age, mean (SD), year	50.7 (11.4)	54.9 (11.3)^a^	49.5 (11.3)	55.9 (9.6)^a^	50.1 (11.3)	55.5 (10.4)^a^
Age group, n (%)						
35–44 years	403 (39)	211 (24)^a^	519 (45)	161 (15)^a^	922 (42)	372 (19)^a^
45–54 years	236 (23)	173 (20)	236 (20)	282 (33)	472 (21)	455 (24)
55–64 years	224 (22)	279 (32)	253 (22)	425 (40)	477 (22)	701 (36)
65–74 years	169 (16)	205 (24)	156 (13)	206 (19)	325 (15)	411 (21)
Education level, n (%)						
None	312 (30.3)	56 (6.5)^a^	470 (40.4)	217 (20.2)^a^	782 (35.6)	273 (14.1)^a^
1–6 years	445 (43.1)	292 (33.8)	532 (45.7)	406 (37.8)	977 (44.5)	698 (36.0)
7–9 years	252 (24.4)	44 (51.0)	151 (13.0)	402 (37.4)	403 (18.3)	843 (43.5)
>9 years	23 (2.2)	76 (8.8)	11 (0.9)	49 (4.6)	34 (1.5)	125 (6.5)
Hypertension, n(%)	402 (39.0)	474 (54.8)^a^	459 (39.4)	630 (58.7)^a^	861 (39.2)	1104 (56.9)^a^
Diabetes, n(%)	14 (1.4)	33 (3.8)^c^	39 (3.4)	59 (5.5)^a^	53 (2.4)	92 (4.7)^a^
Smoking, n(%)	475 (46.0)	318 (36.8)^a^	43 (3.7)	51 (4.7)	518 (23.5)	369 (19.0)^a^
Alcohol consumption, n(%)	195 (18.9)	273 (31.6)^a^	4 (0.3)	48 (4.5)^a^	199 (9.1)	321 (16.6)^a^
SBP, mean(SD), mm Hg	130.5 (18.7)	140.7 (22.3)^a^	132.0 (23.7)	142.6 (22.2)^a^	131.3 (21.5)	141.7 (22.3)^a^
DBP, mean(SD), mm Hg	81.9 (11.2)	86.2 (13.6)^a^	81.5 (12.5)	85.8 (12.1)^a^	81.7 (11.9)	86.0 (12.8)^a^
BMI, mean(SD), Kg/m^2^	22.5 (2.4)	25.0 (3.4)^a^	23.3 (3.3)	25.4 (3.7)^a^	22.9 (3.0)	25.2 (3.6)^a^

Abbreviation: SD, standard difference; SBP, systolic blood pressure; DBP, diastolic blood pressure; BMI, body mass index.

a T-test or Chi-square test between two surveys, P<0.05.

### Trends in prevalence of overweight and obesity by age and gender between 1991 and 2011

Table 2 indicates that the age-standardized prevalence of overweight significantly increased from 24.5% in 1991 to 42.0% in 2011, and the age-standardized prevalence of obesity significantly increased from 5.7% in 1991 to 19.6% in 2011 (*p*<0.05). The increase in prevalence rates of overweight and obesity in men was greater than that observed in women. The prevalence of overweight and obesity in men was 20.4% and 2.6% in 1991 and 41.2% and 17.8% in 2011; the corresponding prevalence in women was 28.7% and 8.3% in 1991 and 42.3% and 21.0% in 2011.

**Table 2:** The age-specific and sex-specific prevalence of overweight and obesity between 1991 and 2011^a^.

Age group	Men		Women		Total	
	1991	2011	1991	2011	1991	2011
Overweight						
35–44 years	19.4 (15.5, 23.2)	42.2 (35.5, 48.8)^c^	32.6 (28.5, 36.6)	36.6 (29.2, 44.1)^c^	26.8 (23.9, 29.7)	39.8 (34.8, 44.8)^c^
45–54 years	18.6 (13.7, 23.6)	43.4 (36.0, 50.7)^c^	30.5 (24.6, 36.4)	47.5 (41.7, 53.3)^c^	24.6 (20.7, 28.5)	45.9 (41.3, 50.5)^c^
55–64 years	25.0 (19.3, 30.7)	38.8 (33.0, 44.5)^c^	24.5 (19.2, 29.8)	44.0 (39.3, 48.7)^c^	24.7 (20.9, 28.6)	41.9 (38.3, 45.6)^c^
65–74 years	19.5 (13.6, 25.5)	38.5 (31.9, 45.2)^c^	21.2 (14.7, 27.6)	41.7 (35.0, 48.5)^c^	20.3 (15.9, 24.7)	40.1 (35.0, 45.3)^c^
Total^b^	20.4 (17.9, 22.8)	41.2 (37.9, 44.5)^c^	28.7 (23.9, 33.5)	42.3 (39.3, 45.2)^c^	24.5 (22.7, 26.3)	42.0 (39.8, 44.2)^c^
Obesity						
35–44 years	1.7 (0.5, 3.0)	16.6 (11.6, 21.6)^c^	7.1 (4.9, 9.3)	18.6 (12.6, 24.6)^c^	4.8 (3.4, 6.1)	17.5 (13.6, 21.3)^c^
45–54 years	3.4 (1.1, 5.7)	19.7 (13.7, 25.6)^c^	8.9 (5.3, 12.5)	23.1 (18.1, 28.0)^c^	6.1 (4.0, 8.3)	21.8 (18.0, 25.5)^c^
55–64 years	2.7 (0.6, 4.8)	16.7 (12.3, 21.1)^c^	11.1 (7.2, 14.9)	21.2 (17.3, 25.1)^c^	7.1 (4.8, 9.4)	19.4 (16.5, 22.3)^c^
65–74 years	3.0 (0.4, 5.5)	18.5 (13.2, 23.9)^c^	6.4 (2.6, 10.3)	21.8 (16.2, 27.5)^c^	4.6 (2.4, 6.9)	20.2 (16.3, 24.1)^c^
Total^b^	2.6 (1.6, 3.6)	17.8 (15.3, 20.4)^c^	8.3 (6.8, 9.9)	21.0 (18.6, 23.5)^c^	5.7 (4.7, 6.6)	19.6 (17.8, 21.4)^c^

a All values are expressed as % (95% confidence interval).

b Age and sex adjusted using the world standard population by the age groups.

c Chi-square test between two surveys, P<0.05.

### Odd ratios of obesity by age and gender

Table 3 outlines the odds ratios of obesity by age group and gender in 2011, using 1991 as a reference. The prevalence of overweight and obesity increased by 1.2-fold and 3.0-fold overall, respectively; the overweight and obesity rates increased by 1.7-fold and 7.1-fold in men and 82% and 1.9-fold in women. The increase in prevalence of overweight and obesity was greater in men than in women. The greatest increase was observed in men aged 35–44 years, with a 10.3-fold increase in obesity prevalence (*p*<0.001). Concurrently, a large increase in the prevalence of overweight was observed in young and middle-aged men, with an increase of more than 2-fold.

**Table 3:** The age-specific and sex-specific odds ratios of overweight and obesity between 1991 and 2011^a^.

Age group	Men		Women		Total	
	OR^b^	95%CI^c^	OR^b^	95%CI^c^	OR^b^	95%CI^c^
Overweight						
35–44 years	3.04	2.10–4.39	1.20	0.83–1.73	1.81	1.40–2.33
45–54 years	3.34	2.14–5.21	2.06	1.44–2.96	2.61	1.97–3.45
55–64 years	1.90	1.29–2.80	2.42	1.72–3.42	2.20	1.70–2.84
65–74 years	2.58	1.61–4.15	1.80	1.16–2.81	2.63	1.88–3.68
Total	2.72	2.22–3.33	1.82	1.53–8.17	2.23	1.95–2.55
Obesity						
35–44 years	11.25	4.90–25.82	2.98	1.78–5.01	4.23	2.82–6.33
45–54 years	6.97	3.14–15.49	3.07	1.81–5.19	4.25	2.75–6.57
55–64 years	7.27	3.04–17.36	2.16	1.47–3.17	4.98	3.33–7.43
65–74 years	7.46	2.87–19.43	4.08	1.99–8.39	5.23	2.95–9.26
Total	8.06	5.30–12.27	2.93	2.27–3.78	4.04	3.27–5.00

a To reference as 1991.

b indicated age-adjusted Odds Ratio.

c indicated 95% confidence intervals of OR.

### Trends in prevalence of obesity by related risk factors between 1991 and 2011

The prevalence of obesity increased significantly in individuals with different risk factors categories, including education levels, blood pressure categories, diabetes previous history, current smoking situation and alcohol drinking situation over the past 21 years overall. The greatest increase in obesity prevalence appeared among those who consumed alcohol (increased by 8.0-fold). Next, there was a 5.3-fold increase in the prevalence of obesity in illiterate residents (Table 4).

**Table 4:** The age- and sex-adjusted prevalence of obesity by cardiovascular risk factors subgroup between 1991 and 2011^a^.

	Men		Women		Total	
	1991	2011	1991	2011	1991	2011
Education level:						
None	2.6 (0.9, 4.3)	3.0 (0, 7.4)	5.9 (3.8, 8.0)	34.2 (27.9, 40.5)^b^	4.6 (3.1, 6.0)	28.8 (23.4, 34.1)^b^
1–6 years	1.4 (0.3, 2.5)	20.0 (15.5, 24.6)^b^	10.0 (7.4, 12.5)	21.8 (17.7, 25.8)^b^	4.7 (3.4, 6.1)	20.5 (17.5, 23.5)^b^
7–9 years	3.8 (1.5, 6.2)	20.3 (16.5, 24.0)^b^	4.0 (0.9, 7.2)	19.2 (15.4, 23.1)^b^	4.2 (2.2, 6.1)	20.8 (18.0, 23.5)^b^
>9 years	4.8 (0, 13.5)	18.2 (9.5, 26.8)	3.4 (0, 14.1)	9.1 (1.0, 1 7.1)	4.1 (0, 10.8)	17.0 (10.4, 23.6)
Hypertension						
No	0	5.8 (1.0, 10.5)	4.0 (1.5, 6.5)	6.8 (1.2, 12.3)^b^	2.4 (0.8, 4.0)	47 (1.5, 7.9)^b^
Yes	4.3 (2.3, 6.3)	24.1 (20.2, 27.9)^b^	13.1 (10.0, 16.2)	25.9 (22.5, 29.4)^b^	9.3 (7.3, 11.2)	25.8 (23.2, 28.4)^b^
Blood pressure Categories:						
Prehypertension	1.5 (0.4, 2.5)	13.5 (9.6, 17.4)^b^	7.2 (4.9, 9.5)	16.2 (12.5, 20.0)^b^	4.1 (2.8, 5.4)	15.2 (12.5, 17.9)^b^
Stage I Hypertension	3.6 (1.4, 5.8)	17.4 (12.5, 22.3)^b^	10.7 (7.0, 14.5)	23.0 (18.6, 27. 4)^b^	7.2 (5.0, 9.4)	20.8 (17.5, 24.2)^b^
Stage II Hypertension	6.2 (1.9, 10.5)	32.3 (26.5, 38.1)^b^	18.3 (12.8, 23.8)	29.6 (24.3, 34.4)^b^	14.4 (10.5, 18.3)	30.9 (27.0, 34.9)^b^
Diabetes						
No	2.5 (1.6, 3.5)	17.8 (15.2, 20.4)^b^	7.7 (6.1, 9.2)	20.4 (17.9, 22.9)^b^	5.2 (4.3, 6.2)	19.2 (17.4, 21.0)^b^
Yes	5.8 (0, 18.0)	27.1 (11.9, 42.3)^b^	23.2 (10.0, 34.5)	55.4 (42.7, 68.1)^b^	19.4 (8.8, 30.0)	39.4 (29.4, 49.4)^b^
Current smoking						
No	2.8 (1.4, 4.2)	21.8 (18.3, 25.2)^b^	8.3 (6.7, 9.9)	20.7 (18.3, 23.2)^b^	6.5 (5.3, 7.6)	20.9 (18.3, 22.9)^b^
Yes	2.4 (1.0, 3.7)	13.1 (9.4, 16.8)^b^	8.5 (0.1, 16.8)	55.3 (41.7, 69.0)^b^	3.0 (1.6, 4.5)	15.6 (11.9, 19.3)^b^
Alcohol Drinking						
No	2.6 (1.6, 3.7)	19.0 (15.9, 22.2)^b^	8.4 (7.8, 11.0)	20.9 (18.4, 23.3)^b^	6.0 (5.0, 7.0)	20.2 (18.3, 22.2)^b^
Yes	2.3 (0.2, 4.4)	17.8 (13.3, 22.3)^b^	0	23.1 (11.2, 35.0)	2.1 (0.1, 4.2)	18.8 (14.5, 23.1)^b^

a All values are expressed as % (95% confidence interval).

b Chi-square test was used to compare the difference between two surveys, P<0.05.

## Discussion

Recent studies have documented that obesity is an independent risk factor for many chronic conditions, resulting in not only hypertension, diabetes mellitus, hyperlipidemia, and metabolic syndrome, but also in an increased incidence of certain cancers [19]–[23].

Since the 1980s, the prevalence of obesity and overweight continues to increase worldwide [1], [24]–[27]. It is predicted that if recent secular trends continue unabated, absolute numbers are projected to reach a total of 2.16 billion overweight and 1.12 billion obese individuals by the year 2030 [28].

The result from the Diogenes cohort showed that the obesity prevalence increased from 13% to 17% since the 1990s [29]. In the United States, the prevalence of obesity increased significantly between 1999 and 2000 (27.5%) and between 2003 and 2004 (31.1%) among men, but not among women (33.4% in 1999–2000 vs. 33.2% in 2003–2004) [30]; however, the increases in the prevalence of obesity do not appear to be continuing at the same rate as that observed between 1999–2000 and between 2007–2008, particularly for women and possibly for men [31].

The data from China indicated that the prevalence of overweight and obesity in men was 9.9% and 0.8% in 1991 and 24.1% and 2.8% in 2002, respectively; corresponding rates in women were 12.9% and 1.9% in 1991 and 26.1% and 5.0% in 2002, respectively [32], [33]. A cross-sectional study conducted in 2004–2006 suggested that the prevalence rates of overweight and obesity in rural China were 20.4% and 2.0%, respectively [34]. In the present study, the prevalence rates of overweight and obesity were 42.0% and 19.6% in 2011, respectively, with respective increases of 1.2-fold and 3.0-fold from 1991. The increase in prevalence of overweight and obesity was greater among men than among women, increasing by 1.7-fold and 82% for overweight and 7.1-fold and 1.9-fold for obesity, respectively. The largest increase was observed in men aged 35–44 years, increasing by 2-fold for overweight and 10.3-fold for obesity.

The previous studies have indicated that obesity is an important risk factor for hypertension and diabetes [35]–[38]; low education level, alcohol consumption, and lack of physical activity are all associated with obesity [9], [39]. Consistent with these studies, in the present study, the prevalence of obesity increased significantly from 1991 to 2011 in individuals, both men and women, who smoked or consumed alcohol, and in those with a lower educational level, hypertension, or type 2 diabetes. The prevalence of obesity among those who consumed alcohol had the greatest increase (increased by 8.0-fold). Moreover, there was a 5.3-fold increase in the prevalence of obesity in illiterate residents overall.

Rapid economic development in China may be a potential explanation for the upward trend in prevalence rates of overweight and obesity observed in our study. The marked increase in obesity prevalence may result in a greater burden on cardiovascular health. Thus, the relationship between obesity and cardiovascular health can never be overestimated.

Over the past 21 years, there has been an extensive shift in the level of agricultural mechanization, resulting in a significant decrease in the need for rural laborers. Furthermore, changes in lifestyle and diet have significantly altered traditional diet and structure among rural residents. Documents from the Ministry of Health of the People’s Republic of China showed that the energy ratio obtained from cereal decreased 15.3%, but the energy ratio obtained from animals and fat increased by 87.1% and 48.9%, respectively, among rural residents from 1992 to 2002 [5]. Moreover, the prevalence of obesity among children and adolescents has risen significantly in China during 1985–2010; in particular, the prevalence rate of obese or overweight increase annually by 9% in boys from rural areas [40], [41]. If this trend continues, this in turn will result in a higher prevalence of hypertension and an increased incidence of cardiovascular disease and stroke.

Our study has several limitations. First, there were certain differences between the 1991 and 2011 surveys with regard to the distribution of subjects’ age and sex (i.e., the number of subjects in the 35–44-year age group was lower in 2011 than in 1991). However, to compare differences in the prevalence of overweight and obesity by education level, hypertension, diabetes, current smoking, alcohol drinking between 1991 and 2011, we adjusted for both age and sex using the world standard population in 2000 as the reference population. In addition, the impact of aging may be more severe in rural areas than in urban areas, as young people migrate from rural areas to cities for economic opportunities or education. Over the past 20 years, the proportion of the Chinese population living in rural areas has decreased from 73% in 1990 to 50% in 2010, whereas only 22% of the population resided in Tianjin, China in 2010. Moreover, the percentage of residents in Ji County aged 35–44 years decreased from 40% in 1991 to 22% in 2011. Therefore, we conducted subgroup analyses stratified by age and sex in the statistical analyses. Second, our data were derived from two cross-sectional surveys conducted in 1991 and 2011 that were used to analyze 20-year trends in the prevalence of overweight and obesity. However, this time interval was relatively long; thus, our study lacks data on 5-year, 10-year, and 15-year trends. Finally, with the exception of smoking and alcohol consumption, we did not have detailed information regarding lifestyle and dietary habits; therefore, other possible determinants of overweight and obesity were not included in this study.

## Conclusions

We assessed the prevalence of overweight and obesity among rural residents of Tianjin, China, and revealed a sharp upward trend following 20 years of rapid economic development. The increase in the prevalence of overweight and obesity was especially significant among both young men and women. The prevalence of obesity increased dramatically among individuals with a low education level and among those who consume alcohol. Our study provides evidence of the severity of the obesity epidemic in adults of rural northern China and the emerging need to control this epidemic. The findings of this study also suggested that obesity is the crucial public health issue in both urban and rural populations in China. In addition, our findings forecast the persistent increase in the incidence of stroke and cardiovascular disease unless these risk factors are efficiently controlled in young adults.
